# Personality disorders (PD) and interpersonal violence (IV) during COVID-19 pandemic: a systematic review

**DOI:** 10.1186/s12991-022-00388-0

**Published:** 2022-04-09

**Authors:** Ramona Di Stefano, Angelica Di Pietro, Dalila Talevi, Alessandro Rossi, Valentina Socci, Francesca Pacitti, Rodolfo Rossi

**Affiliations:** 1 Department of Biotechnological and Applied Clinical Sciences (DISCAB), University of L’Aquila, Via Vetoio, Coppito, 67100 L’Aquila, Italy; 2 Department of Systems Medicine, University of Rome Tor Vergata, Via Montpellier, 1, 00133 Rome, Italy

**Keywords:** COVID-19, Pandemic, Violence, Personality, Disorder, Lockdown, Review

## Abstract

Not only did the ongoing CoronaVIrus Disease-19 (COVID-19) pandemic cause a massive number of casualties, but also there is growing concern that the burden of its psychological aftermaths will only show up years down the road. This systematic review summarises the existing literature reporting the impact of COVID-19 on personality disorders (PDs)-related violence. MEDLINE and APA PsycINFO were independently searched for relevant studies by two authors. Eligible studies had to be identifiable through database searching, published and fully accessible. This systematic review was conducted according to Preferred Reporting Items for Systematic Review and Meta-Analyses (PRISMA) guidelines. An initial pool of 241 studies were found, out of which 69 met the selection criteria and were, therefore, included. The majority reported a worse Mental Health Outcome (MHO) during the pandemic as related to dysfunctional personality and positive personality traits predicting a better outcome. Furthermore, increased levels of interpersonal violence (IV) and self-directed violence were reported. Further research should be conducted on the reciprocal interaction of PDs and IV during the time of pandemic. Nevertheless, the dramatic impact of restrictive measures on PDs has still to be appropriately addressed.

**Supplementary Information:**

The online version contains supplementary material available at 10.1186/s12991-022-00388-0.

## Introduction

The RNA virus Severe Acute Respiratory Syndrome–Corona Virus 2 (SARS–CoV2) is the etiological agent of Coronavirus-2019 disease (COVID-19). On 11 March 2020, COVID-19 was declared a pandemic by the World Health Organization (WHO), affecting economic and health organizations, social relations, and mental health [1]. WHO updated data report 280 million cases and 5.4 million deaths [2].

While dramatic mental health outcomes (MHO) were observed in people affected by COVID-19 [3], restrictive measures have inevitably led to psychopathological consequences in the general population and health care workers [4–6]. Daily habits have changed to face strict prevention measures, such as lockdown, quarantine, or social distancing. Past literature reports that pandemic and related measures affect MHO of those undergoing them [7–9]. Timely studies concerning the first phase of the COVID-19 outbreak reported a substantial psychological impact among the population, suggesting high levels of psychopathological symptoms [5, 10]. People experienced considerable psychological distress during the initial stage of the COVID-19 outbreak in terms of anxiety [5, 10, 11], depression [5, 10], post-traumatic symptoms [5, 10–12] and insomnia [5, 11, 12].

Mounting evidence published during the months after the beginning of the pandemic has confirmed that MHO will persist for longer and peak after the actual pandemic [13]. As a matter of fact, during epidemics, the number of people whose mental health is affected tends to be greater than the number of people affected by the infection itself [14]. Therefore, it appears likely that there will be a substantial increase in behavioral disorders, in terms of externalizing and internalizing symptoms, loneliness, domestic violence, child abuse and drug abuse [9, 15].

Anxious and depressive disorders in the time of pandemic have been extensively investigated [10, 11], while less attention was paid to personality disorders (PDs) and related behavioral disturbances. The link between violence, PDs and dysfunctional personality traits has been described in previous studies [16, 17].

Nevertheless, to our best knowledge, literature lacks a comprehensive study on interpersonal violence (IV) as a domain of PDs interpreted as a possible related outcome of COVID-19 lockdown measures, social activity deprivation and interpersonal functioning modification. The presence of a PD trait or diagnosis seemed to increase the risk of perpetuating or being a victim of violence, both hetero and self-directed [18, 19]. Higher prevalence of PDs diagnoses, and dysfunctional personality traits can be found in violent offenders. In a study by Yu and colleagues, PDs reported a threefold risk of violence than the general population [20]. In light of the aforementioned findings, it is safe to assume that pandemic-related measures would promote and accentuate stress levels and psychopathological severity in individuals with a certain PD or dysfunctional personality trait, in turn raising the chance to perpetuate both hetero and self-directed violence in this population.

This systematic review aimed at summarizing the existing literature reporting the impact of COVID-19 on PD-related violence.

Nonetheless, in a preliminary analysis, no study was found concerning this topic as a *unicum*, i.e., the effects of the COVID-19 pandemic and restrictive measures on PDs and IV taken together. For this reason, the investigation was shifted towards the effect of the pandemic on PDs and violence individually, in hope to shed some light on both areas facilitating future investigation to better understand their connection.

## Methods

### Article identification and inclusion/exclusion criteria

The search period was from 1 January 2020 to 23 May 2021 and was reported according to the 2020 Preferred Reporting Items for Systematic Reviews and Meta-Analysis (PRISMA) guidelines [21].

MEDLINE (National Library of Medicine Bethesda MD) and APA PsycINFO databases were searched through the combination of terms relating to COVID-19, personality disorders and violence. A detailed version of the search line as processed by the search engines is available in Additional file 1: S1. After a preliminary search on other databases, a great redundancy in results was found. Therefore, we limited the systematic search to the aforementioned databases in accordance with PRISMA guidelines [21].

To be eligible, studies had to be identifiable through database searching and fully published. We did not restrict the search to any language. Unpublished and preprint studies were not included. Abstracts and discussion articles were considered eligible. Case reports and case series were included if relevant. Studies regarding pediatric populations were considered beyond the purpose of this review.

A further manual search was conducted on the same databases (MEDLINE and APA PsycINFO) to identify relevant papers, possibly missing from the initial systematic search [22]. Six additional studies were identified through manual search and were reported below. Relevant extracted data was stored in a computerized database.

### Data extraction

Extraction of relevant data was performed independently by RDS and ADP. Disagreement in selection by the two reviewers was solved by consulting DT and VS. AR and RR then approved selected articles. After appropriacy verification, data extracted from the literature were then entered into a standardized form and stored in a computerized database. Abstracted information included first author name, journal, date of publication, study design, study objectives, sample size (if applicable), scales (if applicable), main findings concluded by included articles. No automatic tool was used during any phase of the present study’s selection, review, summarization, or writing.

### Quality assessment

The quality of observational studies was evaluated independently by RDS and ADP using Newcastle Ottawa Scale (NOS). Discrepancies in selection were solved by confrontation. Studies found to be non-satisfactory according to NOS tool were excluded.

## Results

Through the initial search, a total of 241 publications were obtained. Specifically, we obtained 188 studies through the internet database MEDLINE and 53 studies through the internet database APA PsycInfo. Eventually, 69 studies were included concerning the influence of COVID-19 lockdown and pandemic on PDs and violence, both interpersonal and self-direct.

An additional manual search identified six studies. These were not included in the systematic review but were extracted to enrich the systematic search.

Included studies and main extracted data are graphically available in Additional file 2: S2.

**Fig. 1:**
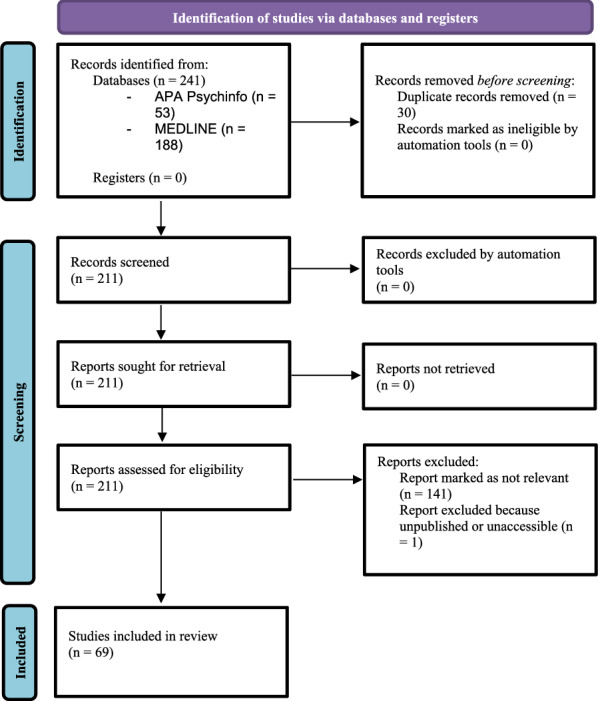
PRISMA flowchart of included studies

A detailed description of selection process is represented in Fig. 1.

Search was focused on the influence of the COVID-19 pandemic on two domains: personality disorders/traits and interpersonal/self-directed violence. An additional group of results was retrieved by manual search.

For the first domain, 23 studies were retrieved about the interaction of COVID-19 pandemic and/or lockdown on PDs and dysfunctional personality traits. Out of these, 15 were observational studies; two were reviews.

Three cross-sectional studies focused on the effect of PDs on adherence to containment measures and the emotional response to COVID-19, finding that subjects with increased psychopathy traits and low empathy tended not to adhere to containment measures [23]. Koich Miguel et al. confirm similar findings, showing how lower adherence to containment measures is found more frequently in subjects with antisocial traits, such as lower empathy and higher callousness, deceitfulness, and risk-taking behavior [24]. It is worth noting that results from these two studies proved the intuitive predisposition to non-cooperation by subjects with antisocial traits. That should be taken into account when designing a public health campaign relying on cooperation, and therefore, subject empathy [24].

Several studies focused their attention specifically on Borderline Personality Disorder (BPD).

In their narrative review, Preti et al. described how the pandemic influenced cluster B PDs, rendering them more susceptible to risky behaviors and impulsiveness. Furthermore, BPD patients showed increased fear of abandonment and difficulty in regulating emotions. Impulsiveness, disregard of others and grandiose self-perception were found to be risk factors for non-compliance to pandemic-related measures [25].

Alvaro et al. gathered data from a 50 BPD patient cohort, examining their clinical course during quarantine. In their study, no significant clinical severity modification was found. Nevertheless, living alone predicted a worse clinical course [26]. Accordingly, in a case report by Chong, the exacerbation of BPD patients sense of isolation, emptiness and fearfulness of social rejection was analyzed as linked to public health measures during coronavirus outbreak [27].

Salamin et al. analyzed an 8-week daily diary, gathering the type and number of episodes of problematic behaviors (*n* = 69), of a small cohort of BPD patients (*n* = 7) undergoing an isolation period during the COVID-19 pandemic. Daily analysis of the results showed how isolation led to a significant reduction in fear, shame, guilt, binge-eating behaviors and overall tension, though their general distress increased [28].

Interestingly, BP symptomatology also increased in chronic pain patients during pandemic [29].

In the aforementioned study by Preti et al., the authors analyzed the effect of the pandemic on other clusters of PDs. In Cluster A PDs, the tendency to introversion facilitates adherence to mitigation measures while worsening their social withdrawal and isolation; Cluster C patients may face difficulties coping with anxiety and fear of contagion. Some of these patients might show higher compliance to restrictive measures, while others may not adhere due to their inflexibility and rigidity, such as in the case of patients affected by Obsessive–Compulsive Personality Disorder [25].

Furthermore, Andrade pointed out a correlation between the Paranoid, Schizotypal and Narcissistic PDs and proneness to believe in COVID-19-related conspiracy theories [30]. Seemingly, Preti et al. highlighted the same analogy regarding Paranoid PDs [25].

For what concerns dysfunctional personality traits, three studies evaluated their possible role in predicting Post-Traumatic Stress Disorder (PTSD) symptoms during COVID-19 outbreak. A commentary study conducted by Coleman highlighted the fact that individuals with higher levels of narcissistic traits may be at greater personal risk for poor trauma-related outcomes, such as PTSD [31]. Similarly, Zhu et al. reported negative emotions and alienation as predictors for PTSD symptoms [32]. Furthermore, in a study conducted by Velotti and colleagues in a cohort of 308 subjects, dysfunctional personality levels, evaluated by Personality Inventory for Diagnostic and Statistical Manual of mental disorders (DSM)-5 “PID-5”, longitudinally predicted PTSD symptomatology [33].

Tommasi et al. described how higher levels of extraversion, agreeableness, conscientiousness, emotional stability, and openness reduced anxiety and depression levels in a sample of 418 responders to an online survey during the first lockdown in Italy [34].

Seemingly, Besser et al. reported lower dependency, self-criticism, fear of not mattering as associated with higher self-reported adaptability to pandemic [35].

Somma et al. studied a cohort of 304 responders (75% females) to an online questionnaire including the 36-item Personality Inventory for DSM-5. In their study, authors proved how Neuroticism-Negative Affectivity could significantly predict clinically relevant depression or anxiety symptoms. In this cohort, dysfunctional personality domains were predictors of worse symptoms, while the psychoticism domain specifically was a significant predictor of clinically relevant acute stress [36]. This may be due to the dysfunctional cognitive and perceptual process characterizing the psychoticism domain, rendering the subject vulnerable to clinically relevant acute stress after lockdown [36].

In a study conducted on 1207 pregnant women during pandemic, Gamache and colleagues found a significant association between levels of personality functioning and affective, behavioral and thought problems [37].

Temperament played an important role in determining the extent of psychological stress during the early pandemic phase, as Moccia et al. reported in a cross-sectional observational study including 500 subjects. In this cohort, cyclothymic, depressive, and anxious temperament and the “needy for approval” attachment style were predictors of moderate-to-severe psychological stress [38].

One study investigated the influence of pandemic on hospital personnel. Ranieri and colleagues focused on the effect of COVID-19 on nurses working frontline on the pandemic. In this study based on 69 nurses, the prolonged exposure to the stressor was progressively faced by personality traits, acting as mediators for the stressor. Furthermore, the agreeableness type of personality was correlated to the short-term development of anxiety symptoms [39].

Examining the influence of the COVID-19 pandemic on PDs patients’ referrals and in-patient admissions to acute mental care facilities, Abbas et al. reported how patients with PDs had overall lower referrals during lockdown (0% of 2020 vs 5% in 2019). This was especially relevant for admissions of emotionally unstable PD patients, showing a sensible reduction in diagnosis rate (6% in 2020 vs 15% in 2019) [40]. In addition, outpatient visits declined for patients diagnosed with PDs, as found by Giannouchos and colleagues [41]. On the contrary, Seifert and colleagues reported an increase of repeated visits in the Psychiatric Emergency Department within 1 month from the first visit among PD patients [42].

Finally, different studies explored the therapeutic options for PDs and their role in coping with the pandemic. In their cross-sectional study based on data gathered from 28 therapists, Lakeman and colleagues described the role of Dialectical Behavioral Therapy in treating BPDs during the pandemic, finding that BPD patients were more prone to clinical deterioration and more prone to access to emergency services [43]. Ventura Wurman et al. examined the role of Mentalization Based Therapy in handling emotion dysregulation in BDP patients during the pandemic. In the study, the authors emphasized how interventions have to be arousal-adjusted to decrease arousal and help re-instate mentalizing [44]. In the attempt to evaluate the role of online interventions targeting PD patients, Reis et al. identified the characteristics of an effective online intervention, including presence of designed modules and materials, a psychoeducational approach and a method to assess patients’ progresses [45].

The second focus was represented by the influence of COVID-19 lockdown/pandemic on IV and self-direct violence. For this topic, 46 eligible studies were identified. Out of these, 32 observational studies and 5 reviews were included. No randomized controlled trial was found.

Self-isolation, social distancing, limited access to help lines, low income and unemployment, psychiatric disorders are some of the risk factors related to Intimate Partner Violence (IPV) which were found to increase during the COVID-19 pandemic [46, 47]. In addition, higher anxiety was found to be associated with higher total abuse scores during the pandemic in a cohort of 172 responders to a survey [48]. In addition, the vulnerability of stalking victims was found to be increased during lockdown [49].

Investigating the impact of COVID-19 on frequency and type of violence perpetrated, Piquero and colleagues reported an increase in Domestic Violence (DV) during the first 2 weeks of lockdown and a reduction thereafter [50].

As regarding the general population, different studies reported an increase in experienced verbal, physical or sexual IPV and DV during lockdown [51–57].

Seemingly, in a study performed by Every-Palmer et al., 10% of participants reported the presence of violence from family members during the lockdown. Furthermore, 6% reported suicidal ideation and 2% suicide plans or attempts [58]. In addition, the severity of IPV increased [59]. On the contrary, Tierolf et al. found no difference in violence perpetrated in families before and after lockdown [60].

Alcohol consumption and unemployment were found to be sources of motivation for DV perpetration during lockdown [61].

Regarding Violence Against Women (VAW), several studies reported an increase in emotional, verbal and physical violence during the pandemic [62–64]. In addition, an exacerbation of the already known risk factors for VAW, such as pregnancy, young age and being a migrant, was reported [65]. In a study by Endler et al., based on an online survey with 51 responders, 79% reported an increase in the risk of gender-based violence and sexual violence [66]. Seemingly, a survey conducted on 687 Jordanian women showed that 40% of participants experienced violence. Being married and unemployed was found to be a significant predictor of VAW [67].

Interestingly, no increase in the absolute number of emergency room accesses for VAW in the lockdown period was reported [68].

Di Franco and colleagues administered an ad hoc questionnaire to those patients accessing Emergency Department (ED) to ascertain the frequency of DV cases during the lockdown in a hospital in South Italy. This questionnaire contained an adapted version of the “Multi-country Study on Women’s Health and Domestic Violence against Women”. Of those completing the questionnaire, 22.7% disclosed a recent history of DV, while those not participating in the survey reported DV in 0.6% of cases [69].

Interestingly, a study reported how parents perceived the lockdown-associated isolation as correlated with an increase in spanking and discipline [70].

Furthermore, lower income, the presence of adverse childhood experiences and hostile sexism seemed to correlate with the risk of DV in the containment measure period [71, 72].

Two studies showed the influence that COVID-19 lockdown had on violence patterns, visibility of harmful behaviors and the inadequacy of support system to face this violence outbreak [73, 74]. Barbara and colleagues showed a reduction in the number of women seeking help at the Service for Sexual and Domestic Violence during home confinement [75]. Accordingly, other studies found a reduction in reporting DV and seeking help during lockdown [53, 76–79]. Conversely, two studies reported an increase in the rate of calls to help lines for DV [80, 81], and the successful use of telemedicine in preventing and detecting violence cases [81]. No increase in emergency calls to help lines due to DV was reported in a study by Gil-Jardiné [82].

Several studies observed ED accesses for reasons related to violence during the lockdown period to analyze relevant tendencies and patterns. Olding et al. reported IV and Deliberate Self Harm (DSH) as the most frequent causes for ED admission during the SARS-CoV2 pandemic. Trauma-related access to ED due to DSH raised in proportion during 2020 compared to data from 2019 [83, 84].

In a retrospective observational study analyzing ED accesses during the lockdown, a reduction in the overall assault rate and DV perpetrated by husbands was observed. On the contrary, DV admittances to ED performed by unspecified family members raised (1.7% vs 1.1%) compared to the previous year [85]. Similarly, no differences in accesses due to DSH, assault [86] or IPV [87] was found. In addition, two studies reported a significant reduction in ED admittance due to DV and sexual assault during the lockdown and pandemic [88, 89].

Rates of crimes committed without a perpetrator peer group, i.e., homicide and IPV, remained unchanged or increased during the SARS-CoV2 pandemic [90].

Ghoshal et al. considered COVID-19 and DV a “twin crisis”, underlining the role of stakeholders and researchers in stopping these two emergencies [91].

The third group of studies identified through a manual search led to the identification of six additional studies concerning the topic.

A cross-sectional study on 1319 responders demonstrated how personality factors significantly correlate with compliance to restrictive measures, distress, changes in behavior, fears, concerns and beliefs concerning the pandemic. A multiple regression analysis showed how extraversion and contentiousness had a more significant correlation with precaution behaviors to avoid infection. Neuroticism and agreeableness had a more significant relation with COVID-19 impacts on participants’ concerns, fears, opinions, and beliefs. In addition, higher neuroticism and lower agreeableness scores were associated with more distress and negative behaviors due to COVID-19 [92].

Similarly, Gori et al. highlighted the association between Big Five Personality traits, coping mechanisms and the aftermath of events during the pandemic. Agreeableness and conscientiousness were found to favour a more functional coping mechanism while being associated with the presence of post-traumatic symptoms. When investigating neurotic traits, the opposite was found to be true [93]. Accordingly, in a study by Shokrkon et al., extraversion was associated with higher emotional, psychological and social wellbeing than neuroticism [94].

An observational study based on 604 participants linked the Dark Triad (psychopathy, narcissism and Machiavellianism) of personality to compliance with pandemic restrictions and the presence of anxiety and depressive symptomatology. Researchers found a possible relationship between higher levels of psychopathy and a reduction in compliance with COVID-19 Pandemic recommendations. In addition, the intensity of the Dark Triad correlated with an increased occurrence of depressive symptomatology. Interestingly, higher levels of subclinical narcissism seem to contribute to civil compliance and increased anxiety symptoms [95]. On the same note, Doerfler et al. investigated the role of the Dark Triad traits in engaging risky decision-making during the COVID-19 pandemic. Psychopathy emerged as a significant predictor of risk-taking behaviors during the current pandemic crisis [96]. Furthermore, a study conducted by Zajenkowski et al. found reduced compliance to containment measures as associated with higher psychopathy, narcissistic and Machiavellianism levels [97].

Given the great number of findings, we decided to analyze the studies included in this review according to an “expectation criterion”, i.e., grouping the manuscripts in those proving intuitive results or results in line with previous literature, results reporting no change from pre-COVID-19 literature and those reporting partially/counter-intuitive results. This subdivision is highlighted in Table 1.

**Table 1:** Graphical representation of findings about PDs and violence as a function of intuitiveness

Intuitive results	Neutral results	Partially/Counter-intuitive results
*Findings about PDs as a function of intuitiveness*		
a. PDs or dysfunctional personality traits increasing the risk of negative outcomes [23–33, 36, 38, 42, 92–97] b. Functional (positive) personality traits leading to positive outcomes [34, 35, 37, 92–94]	No change in clinical severity [26]	Decline in hospital visits [40, 41] Anxiety was correlated to agreeableness in hospital personnel [39]
*Findings about Violence as a function of intuitiveness*		
Findings confirming an expected outcome [46–49, 51–59, 61, 63–68, 70–74, 80–84, 89, 91]	Findings reporting no change during COVID-19; Findings not discussible in terms of expected outcome [60, 68, 79, 82, 86, 87, 90]	Findings demonstrating the opposite of an expected outcome [69, 75, 76, 78, 85, 88, 89]

The correspondent reference number may appear in multiple categories when multiple findings were reported on the same study

## Conclusions and discussion

This systematic literature review examined the effects of the COVID-19 pandemic and restrictive measures on personality disorders and violence patterns.

Retrieved literature showed the negative impact of the COVID-19 pandemic on PDs in terms of clinical severity, coping with emotions and stress levels [25–28] and how coping strategy efficacy is directly correlated to a positive psychopathological outcome, especially for personality disorder patients [34, 35, 37, 39]. This, in turn, depends on treatment choice, follow-up during quarantine and positive personality traits, such as agreeableness and conscientiousness [34, 39, 43, 44].

Nevertheless, literature proved insufficient in properly determining the psychopathological outcome of personality disorder patients during the pandemic.

Furthermore, few investigations were conducted to find the most efficacious approach/treatment to manage the acute complications that these patients may suffer during a period, such as a pandemic, especially during self-isolation [43–45]. Similarly, insufficient literature is available about the reported or possible role of telemedicine and telepsychiatry in dealing with emergencies such as this.

For what concerns the effects of COVID-19 on IPV, literature revealed increased levels of both hetero and self-directed violence [50, 51, 53–55], while report rates and supporting network seeking were found to decrease during the pandemic [53, 75–79]. Mitigation measures highlighted the need for improvement of support services for IPV victims.

Previous findings showed how PDs are more prone to act as perpetrators of both hetero and self-directed violence [98, 99]. Together with the difficulties in coping with the lockdown suffered by these patients, these findings may lead to the assumption of an increase in violence rate during lockdown periods among PD patients (i.e., ***Intuitive results***, see above). Therefore, further research should be aimed at confirming such hypothesis.

While the majority of findings are in line with previous literature, we were drawn to those proving the opposite of previous literature or a deviation from what was reasonably expected. This is the situation of Giannouchos et al. and Abbas et al., both reporting a reduction in hospital visits by personality disorder patients and referrals in ED. These findings could be explained by a possible shift of the locus of care, a fear of contagion or, as authors point out, by an improvement of telemedicine intervention [40, 41].

As demonstrated by most of the literature retrieved in this review, dysfunctional and negative personality traits and PDs were positively correlated to negative outcomes in coping with the pandemic, as well as functional and positive personality traits predicted better MHO. Contrarily, Ranieri et al. reported the agreeableness type of personality as a mediator of the short-term acute stress in frontline nurses, probably due to the young age of the cohort, the lack of psychological support and the sudden onset of the stressor [39].

As for COVID-19 influence on IPV, a counter-intuitive reduction in assault rates, access to ED for violent reasons, decrease in seeking help and abuse reporting were described by multiple researchers [69, 75, 76, 78, 85, 88, 89]. These phenomena could be explained by the difficulty of physically exiting the house for fear of being discovered by the abusive partner or fear of contagion during the overt pandemic. In addition, a decreased report rate has to be noted when receiving data from online surveys, as victims may not access a secure environment for their completion. At the same time, the overall reduction in access to ED due to street crimes, such as robbery and assault, could be easily explained by home confinement, decreased access to drugs and alcohol, or simply by a decreased ability to meet the assaulter.

Research on the effect of the COVID-19 pandemic and restrictive measures on PDs reported inhomogeneous results. This may be due to the scarce literature on this topic. In addition, it should be taken into account that heterogenicity in results may be the effect of a wide array of study designs and the difference in environmental conditions in which results were gathered.

Few studies focused on evaluating the effectiveness of specific interventions or treatment for PD patients, reflecting the lack of findings on the topic.

An important obstacle to overcome is the difficulty in finding an appropriate and uniform way to gather sufficient data, since this would require an enormous effort. This issue justified the non-assisted method of data gathering through online surveys and similar, leading to uncontrolled results. Nevertheless, we hope the present systematic review was capable to bring the attention to the role of the pandemic on PD clinical condition and worldwide IV increase.

## Future implications

Further research should be conducted on the reciprocal interaction of PDs and IV during the time of pandemic. For example, it could be useful to demonstrate how the perpetration of violent patterns might hide behind the curtain of the worsened psychopathological state experienced by PD patients and how much of the increased violence during the pandemic may be linked to levels of dysfunctional personality traits or PDs.

Furthermore, research should address the impact of public health policies, such as the restrictive measures on PDs specifically, trying to overcome the lack of data concerning this subset of patients.

## Strengths and limitations

Strengths of this review include the systematic approach of data collection, allowing for a more reliable result analysis, the consistent amount of literature retrieved and the quality assessment of included observational studies performed by means of standardized international scales (NOS). Furthermore, an additional manual search was performed to enrich the study with evidence otherwise lost.

Limitations to the present study may be represented by the inclusion of studies based on non-assisted or not-standardized data gathering, information retrieved during different pandemic periods and involving countries with different restrictive measures and cultures.

## Supplementary Information

**Additional file 1: S1.** Search Line Syntax.

**Additional file 2: S2**. Included studies and main extracted data.

## Data Availability

All data generated or analyzed during this study are included in this published article [and its additional information files].

## Abbreviations

SARS–CoV2: Severe Acute Respiratory Syndrome–Corona Virus 2
COVID-19: CoronaVIrus Disease-19
WHO: World Health Organization
MHO: Mental Health Outcomes
PD: Personality Disorder
IV: Interpersonal violence
PRISMA: Preferred Reporting Items for Systematic Review and Meta-Analyses
NOS: Newcastle Ottawa Scale
BPD: Borderline Personality Disorder
PTSD: Post-Traumatic Stress Disorder
DSM: Diagnostic and Statistical Manual of mental disorders
IPV: Intimate Partner Violence
DV: Domestic Violence
VAW: Violence Against Women
ED: Emergency Department
DSH: Deliberate Self Harm
