# Supplementary material for: Personality disorders (PD) and interpersonal violence (IV) during COVID-19 pandemic: a systematic review
Source: Ann Gen Psychiatry. 2022 Apr 9;21:11. doi: 10.1186/s12991-022-00388-0 (PMC8994418; doi:10.1186/s12991-022-00388-0)
Supplement: Supplementary file 1 — Additional file 1: S1. Search Line Syntax. [file 12991_2022_388_MOESM1_ESM.docx]

**ADDITIONAL MATERIAL - S1: Search Line Syntax**

**MEDLINE Search Line for “Personality disorder” AND “COVID”:**

*Processed by the database as*:

Search: (personality disorder) AND (covid) Sort by: Most Recent ("personality disorders"[MeSH Terms] OR ("personality"[All Fields] AND "disorders"[All Fields]) OR "personality disorders"[All Fields] OR ("personality"[All Fields] AND "disorder"[All Fields]) OR "personality disorder"[All Fields]) AND ("sars cov 2"[MeSH Terms] OR "sars cov 2"[All Fields] OR "covid"[All Fields] OR "covid 19"[MeSH Terms] OR "covid 19"[All Fields]) Translations personality disorder: "personality disorders"[MeSH Terms] OR ("personality"[All Fields] AND "disorders"[All Fields]) OR "personality disorders"[All Fields] OR ("personality"[All Fields] AND "disorder"[All Fields]) OR "personality disorder"[All Fields] covid: "sars-cov-2"[MeSH Terms] OR "sars-cov-2"[All Fields] OR "covid"[All Fields] OR "covid-19"[MeSH Terms] OR "covid-19"[All Fields]

**MEDLINE Search line for “COVID” AND “Lockdown” AND “violence”:**

*Processed by the database as*:

Search: ((covid) AND (lockdown)) AND (violence) Sort by: Most Recent ("sars cov 2"[MeSH Terms] OR "sars cov 2"[All Fields] OR "covid"[All Fields] OR "covid 19"[MeSH Terms] OR "covid 19"[All Fields]) AND "lockdown"[All Fields] AND ("violence"[MeSH Terms] OR "violence"[All Fields] OR "violence s"[All Fields] OR "violences"[All Fields]) Translations covid: "sars-cov-2"[MeSH Terms] OR "sars-cov-2"[All Fields] OR "covid"[All Fields] OR "covid-19"[MeSH Terms] OR "covid-19"[All Fields] violence: "violence"[MeSH Terms] OR "violence"[All Fields] OR "violence's"[All Fields] OR "violences"[All Fields]

**APA PsycInfo Search line for “Personality disorder” AND “COVID”**

S2 personality disorder AND COVID Expanders - Applica argomenti equivalenti Search modes - Boolean/Phrase Interface - EBSCOhost Research Databases Search Screen - Advanced Search Database - APA PsycInfo 16

**APA PsycInfo Search Line “COVID” AND “Lockdown” AND “violence”**

S1 covid AND lockdown AND violence Expanders - Applica argomenti equivalenti Search modes - Boolean/Phrase Interface - EBSCOhost Research Databases Search Screen - Advanced Search Database - APA PsycInfo 34
