# Supplementary material for: Personality disorders (PD) and interpersonal violence (IV) during COVID-19 pandemic: a systematic review
Source: Ann Gen Psychiatry. 2022 Apr 9;21:11. doi: 10.1186/s12991-022-00388-0 (PMC8994418; doi:10.1186/s12991-022-00388-0)
Supplement: Supplementary file 2 — Additional file 2: S2. Included studies and main extracted data. [file 12991_2022_388_MOESM2_ESM.docx]

| PERSONALITY DISORDERS & LOCKDOWN/PANDEMIC | | | | | | | |
| --- | --- | --- | --- | --- | --- | --- | --- |
| Authors | **Date of Publication** | **Journal** | **Study Design** | **Number of sample (subjects)** | **Tested outcome** | **Scales and measures** | **Main findings** |
| Richard Lakeman et al. | 15/09/2020 | Issues Ment Health Nurs | Cross-Sectional Study | 28 | Role of DBT (Dialectical Behavior Therapy) in treatment of BPD (Borderline Personality Disorder) patients during pandemic | NA | Clinical deterioration and increased use of crisis services in place of DBT programs |
| Lucas de Francisco Carvalho et al. | 22/09/2020 | Trends Psychiatry and Psychotherapy | Cross-Sectional Study | 893 | Relationship between psychopathy traits and containment measures adherence | PID-5 (Personality Inventory for DSM-5); ACME (Affective and Cognitive Measure of Empathy) | Higher psychopathy trait levels and low levels of empathy associated with less adherence to containment measures |
| Antonella Somma et al. | 29/12/2020 | J Affect Disord. | Cross-Sectional Study | 304 | Emotional response to COVID-19 | PID-5-36; DSM-5 level 2 Depression; DSM-5 level 2 Anxiety; DSM-5 Severity of Acute Stress Symptoms | Neuroticism-NA found to be a predictor of clinically relevant depression and anxiety; Psychoticism predictor of relevant acute stress |
| Fabiano Koich Miguel et al. | 01/01/2021 | Pers Individ Dif. | Cross-Sectional Study | 1578 | Antisocial traits and containment measures relationship | PID-5; ACME | Antisocial traits (lower levels of empathy and higher of Callousness, Deceitfulness and Risk-taking) directly associated with lower compliance with containment measures |
| Lorenzo Moccia et al. | 20/04/2020 | Brain Behav Immun. | Cross-Sectional Study | 500 | Psychological distress perceived by Italians during early phase of pandemic | K10 (Kessler 10 psychological distress scale); TEMPS-A (temperament evaluation of Memphis, Pisa, Paris and San Diego-Auto-questionnaire); ASQ (attachment style questionnaire) | Cyclothymic, depressive and anxious temperaments, and the ASQ “Need for approval” as risk factors for moderate-to-severe psychological distress compared to no distress |
| Seng Choi Chong | 23/04/2020 | Asian Journal of Psychiatry | Case Report | 1 | Functioning and responses of BPD patients during pandemic | NA | Patients reported sense of isolation, emptiness, fearfulness of social rejection |
| Hannah Shapiro et al. | 15/06/2020 | Journal of Pain Research | Commentary Study | NA | NA | NA | Comment on increased BP symptomatology in chronic pain patients during pandemic |
| Frias Álvaro et al. | 16/07/2020 | Psychiatry Research | Letter to the editor/Exploratory cross-sectional study | 50 | Clinical course of BPD patients during quarantine | CGI-I (Clinical Global Impression-Improvement) Spanish Version | Absence of a significant clinical severity modification; Living alone predicted worse clinical course |
| Saluminn R M Coleman | 12/08/2020 | Psychol Trauma. | Commentary Study | NA | Potential associations between narcissism and trauma-related outcomes during pandemic | NA | Individuals higher in narcissism may be at greater personal risk for poor trauma-related outcomes such as PTSD |
| Virginie Salamin et al. | 20/08/2020 | Ann Med Psychol (Paris) | Cross-Sectional Study | 7 | Impact of pandemic on BPD patients | NA | Reduced Shame, Guilt, Fear, Tension, Binge-eating Behaviors; Increased distress |
| Gabriel Andrade | 01/09/2020 | Asian J Psychiatr. | Letter to the Editor | NA | Conspiracy theories | NA | Correlations between proneness to believe in conspiracy theories and Paranoid, Schizotypal and Narcissistic Personality Disorders |
| Tamara Ventura Wurman et al. | 07/09/2020 | Counselling Psychology Quarterly | Descriptive Study | NA | NA | NA | Description of BPD patient responses to MBT (Mentalization-based Therapy) in regulating emotions during pandemic |
| Emanuele Preti et al. | 10/11/2020 | Current Psychiatry Reports | Narrative Review | NA | Impact of pandemic on PDs (Personality Disorders) | NA | Cluster B more susceptible to risky behaviors and impulsiveness. BPD patient difficulties in regulating emotions and fear of abandonment |
| Avi Besser et al. | 18/11/2020 | Int J Ment Health Addict. | Cross-Sectional Study | 462 | Relationship between adaptability to COVID-19 pandemic and personality traits | NA | Higher self-reported adaptability to pandemic associated with lower dependency, self-criticism, fear of not mattering |
| Theodoros V Giannouchos et al. | 09/12/2020 | Am J Emerg Med. | Retrospective Cross-Sectional Study | 80,000 ca | Outpatient ED (Emergency Department) visit trends | NA | Decline in visits classified as non-emergent for patients diagnosed with mood and personality disorders |
| Marco Tommasi et al. | 17/12/2020 | Front. Psychol. | Cross-Sectional Study | 418 | Psychological impact of first lockdown | BFO (Big Five Observer Questionnaire); STAI (State-Trait Anxiety Inventory); BDI (Beck's Depression Inventory); LOT-R (life orientation test revised) | High levels of extraversion, agreeableness, conscientiousness, emotional stability and openness linked to reduction in anxiety and depression levels |
| Dominick Gamache et al. | 07/01/2021 | Personal Disord. | Cross-Sectional Study | 1207 | Impact of pathological personality on psychiatric problems in pregnant women during pandemic | Criterion A elements of alternative model for PDs | Significant association between levels of personality functioning and affective/behavioral/thought problems |
| Samantha Reis et al. | 20/01/2021 | Res Psychother | Scoping and Rapid Review | NA | Online intervention targeting PDs | NA | Characteristics of effective online intervention targeting PDs |
| Johanna Seifert et al. | 05/02/2021 | Eur Arch Psychiatry Clin Neurosci. | Cross-Sectional Study | 392 | Effects of pandemic on patients presenting in ED | NA | Increase of repeated visits within one month, especially among PDs patients |
| Yue Zhu et al. | 15/02/2021 | Journal of affective disorders | Cross-Sectional Study | 7145 | Relationship between negative emotions and PTSD | SCL-90 (Symptom Checklist-90); PCL-C (PTSD Checklist-Civilian version); ASAS (Adolescent students Alienation Scale) | Negative emotions and alienation as predictors for PTSD symptoms and their effects mediated by anxiety levels |
| Mohammed J Abbas et al. | 01/03/2021 | Psychiatry Serv. | Retrospective Cross-Sectional Study | NA | Impact of pandemic on mental health | NA | Lower referrals and inpatient admission; fewer patients diagnosed with emotionally unstable PDs (6% in 2020 vs 15% in 2019) and with any other PDs (0% vs 5%) |
| Patrizia Velotti et al. | 23/03/2021 | Frontiers in Psychiatry | Cross-Sectional Study | 308 | Find vulnerability traits linked to PTSD during pandemic | DERS (difficulties in emotion regulation scale); DES-II (Dissociative Experience Scale); PID-5; NSESSS (National Stressful Events Survey PTSD Short Scale); FCV-19S (Fear of COVID-19 scale) | Pathological personality levels longitudinally predicted PTSD and fear of COVID-19 levels |
| Jessica Ranieri et al. | 06/04/2021 | Front Psychol. | Cross-Sectional Study | 69 | Impact of COVID-19 on nurses | BFI-10 (Big Five Personality Inventory); DASS-21 (Depression Anxiety Stress Scales); IES-R (Impact of Event Scale- Revised); PDEQ (Peritraumatic Dissociative Experiences Questionnaire) | Personality traits acted as mediators in facing subjective stress in frontline nurses during COVID-19. Short-term development of anxiety symptoms was correlated to agreeableness type of personality. |

| VIOLENCE & LOCKDOWN/ PANDEMIC | | | | | | | |
| --- | --- | --- | --- | --- | --- | --- | --- |
| Authors | **Date of Publication** | **Journal** | **Study Design** | **Number of Sample** | **Tested Outcome** | **Scales and Measures** | **Main Findings** |
| Alex R. Piquero et al. | 14/06/2020 | Am J Crim Justice. | Cross-Sectional Study | NA | Impact of COVID-19 on number and type of violence reports to police stations | NA | Increase of domestic violence during the first 2 weeks of lockdown, and a reduction thereafter |
| Heather X Rhodes et al. | 26/08/2020 | Cureus. | Retrospective Cross-Sectional Study | 2900 | Impact of lockdown on type and number of accesses to ER (Emergency Room) | NA | Reduction in assault rate (overall); DV (Domestic Violence) by husbands reduced from 33% to 0% over the study period; DV by unspecified family member raised to 1.7% vs 1.1% when compared to the previous year |
| Minakshi Dahal et al. | 21/09/2020 | Global Health. | Commentary study | NA | VAW (Violence Against Women) in Nepal during lockdown | NA | COVID-19 increased the risk of DV and the support system to face the violence against women and young girls (VAWG) was not functional |
| Sophie Joanne Weller et al. | 22/09/2020 | Sex Transm Infect. | Letter to the editor | NA | Increase of DV during lockdown and the role of telemedicine | NA | Increase in percentage of disclosures of DV within an Integrated Sexual Health (ISH) service using mainly telemedicine during lockdown. |
| Vishal Bhavsar et al. | 28/09/2020 | Lancet Psychiatry. | Commentary Study | NA | Link between mental illness and DV | NA | Pandemic and lockdown increased DV cases and reduced the visibility of harmful behavior |
| Susan Jacob et al. | 29/09/2020 | ANZ J Surg. | Cross-Sectional Study | NA | Evaluate the effect of COVID-19 on trauma related accessed to ER | Injury Severity Score (ISS) | No change in ISS, decrease in RTA (road traffic accident), and falls. No difference in accesses due to DSH (Deliberate Self-Harm) or assault |
| Giussy Barbara et al. | 29/10/2020 | J Womens Health | Retrospective Cross-Sectional Study | NA | Increased IPV (Intimate Partner Violence) related to lockdown | NA | Reduction in the number of women seeking help at the SVSeD (Service for Sexual and Domestic Violence) during lockdown |
| Jena Derakhshani Hamadani et al. | 01/11/2020 | The Lancet Global Health | Cross-Sectional Study | 2424 | Immediate impact of lockdown on women and families | NA | Over half of women experiencing emotional or moderate physical violence reported increased DV episodes since the beginning of lockdown |
| Susanna Every-Palmer et al. | 04/11/2020 | PLoS One. | Cross-Sectional Study | 2416 | Assess the psychological well-being of New Zealanders during lockdown | GAD-7 (General Anxiety Disorder), WHO-5, and K-10 (Kessler Psychological Distress Scale) | 30% reported moderate to severe psychological distress; 16% high levels of anxiety; 39% lower wellbeing; suicidal ideation 6%, suicide plans 2% and 2% suicide attempts; 10% violence from family members during lockdown |
| Stefanie Jung et al. | 18/11/2020 | J Clin Med. | Cross-Sectional Study | 3545 | Assessment of mental health in response to lockdown in Germany | PHQ-4 (Patient Health Questionnaire), PHQ stress module, WHO-5 well-being index and SOC-L9 (sense of Coherence Scale-short form Leipzig) | 5% of all participants reported IV (interpersonal violence) on a verbal, physical and sexual level |
| Wajiha Haq et al. | 24/11/2020 | Peer J. | Cross-Sectional Study | 257 | Variables changing severity and type of IPV during lockdown | NA | 35% of women reported IPV during lockdown, 17% faced physical violence during lockdown (up to 12 times); 28% reported verbal violence; 34% emotional violence |
| Amrit Pattojoshi et al. | 01/12/2020 | Psychiatry Clin Neurosci. | Cross-Sectional Study | 560 | Assessment of prevalence and characteristic of DV during lockdown | NA | About 40% of victims do not resort to any safety measure |
| Shawna J. Lee et al. | 14/01/2021 | J Fam Violence. | Cross-Sectional Study | 283 | Effect of lockdown on violence against children | NA | Parents perceived their isolation (lockdown) as correlated with an increase in spanking and discipline |
| Minna Lyons et al. | 26/02/2021 | J Fam Violence. | Qualitative Thematic Analysis | 50 | Analyze the effect of lockdown on severity of IPV by analyzing Reddit posts | NA | IPV found to be more severe during lockdown |
| Kazhan I Mahmood et al. | 26/02/2021 | J Interpers Violence. | Cross-Sectional Study | 346 | Evaluate the effect of lockdown on spousal violence in Kurdistan | NA | Violence increased (+32%) during lockdown, +35% emotional abuse, +29% physical abuse (pulling hair, twisting arms); 9% reported forced intercourse compared to 6% during pre-COVID period |
| Priyanka Sharma et al. | 03/03/2021 | Disaster Med Public Health Prep. | Cross-Sectional Study | 94 | Analyze the effect of lockdown on violence and coping strategies | NA | 8% of interviewed subjects had faced DV during lockdown, half of them ignored the problem or coped using yoga/meditation |
| Rob Stephenson et al. | 06/03/2021 | J Interpers Violence. | Cross-Sectional Study | 696 | Assess increased violence between GBMSM (Gay Bisexual and Other Men who Have Sex with Men) | NA | Higher rates of emotional violence; reporting IPV during lockdown was lower. |
| Vera Clemens et al. | 18/03/2021 | Psychotherapeut (Berl). | Cross-Sectional Study | 687 | Link between IPV and mental health problems in children and adolescents | ACE Questionnaire (Adverse Childhood Experiences) | Lower household incomes and the experience of own adverse childhood experiences increased the risk of participants reporting violence in their relationship |
| Nickola C Overall et al. | 18/03/2021 | J Fam Psychol. | Cross-Sectional Study | 362 | Sexist attitudes role in prediction of DV during lockdown | NA | Males endorsing hostile sexism reported greater aggressive behavior during lockdown |
| Giulio Nittari et al. | 06/04/2021 | Int J Environ Res Public Health. | Prospective and Retrospective Cross-Sectional Study | NA | Evaluation of VAW increase during lockdown | NA | No increase in absolute number of accesses in 2020 but reported increase of severity of injuries |
| Rakhi Ghoshal | 07/05/2020 | Indian J Med Ethics. | Commentary Study | NA | Impact of COVID-19 on DV in India | NA | Two emergencies are described: DV and COVID-19. The limited actions by stakeholders and studies concerning DV are causing themselves an increase in DV |
| James Olding et al. | 30/06/2020 | Surgeon. | Prospective and Retrospective Cross-Sectional Study | NA | Identification of a change in patterns of violence | NA | The main ED admissions were for IV and DSH. IV remained the most common etiology overall if compared with 2019. DSH etiology in 2020 accounted for 27% of causes, compared to 11% in 2019 |
| John H. Boman IV et al. | 08/07/2020 | Am J Crim Justice. | Commentary Study | NA | Impact of COVID-19 on Crime patterns | NA | Decrease in crimes committed in peer groups; Crimes committed without co-offenders, as homicide and IPV, remained either unchanged or increased. |
| Diana Nadine Moreira et al. | 26/07/2020 | Int J Law Psychiatry. | Narrative Review | NA | Impact of COVID-19 on IPV | NA | Identification of risk factors related to IPV that can increase with covid-19: Self isolation and social distancing; limited access to help lines; Low income and unemployment; Rigid gender rules; Psychiatric disorders |
| Hongwei Zhang | 04/09/2020 | J Fam Violence. | Commentary Study | NA | Influence of pandemic on DV | NA | Multiple reports suggest an increase in family violence in China due to lockdown and lack of support for victims |
| Kelly Bracewell et al. | 10/09/2020 | J Fam Violence. | Cross-Sectional Study | 21 | Consequences of lockdown on stalking | NA | Vulnerability of Stalking victims increased. Some restrictions provided increased opportunities for stalkers to monitor their victims |
| Odette R Sánchez et al. | 23/09/2020 | Int J Gynaecol Obstet. | Review | NA | VAW during restrictive measures | NA | Factors increasing vulnerability to violence (pregnant women, migrants, young people etc.) have been exacerbated due to the impact of the pandemic and the social distancing measures on daily life |
| Jorge M Agüero | 29/09/2020 | World Dev. | Retrospective Cross-Sectional Study | NA | Assessment of increased VAW during pandemic | NA | Call incidence rate increased by 48 percent between April and July 2020 |
| Sabrine Sediri et al. | 17/10/2020 | Arch Womens Ment Health. | Cross-Sectional Study | 751 | Impact of pandemic on DV | DASS-21; FBAS (Facebook Bergen Addiction Scale) | VAW increased from 4.4% to 14.8% with psychological abuse as the main type of violence; Women abused before increased risk of violence during lockdown |
| Margit Endler et al. | 11/11/2020 | Acta Obstet Gynecol Scand. | Cross-Sectional Study | 51 | Evaluate the impact of COVID-19 on sex health, sex violence, abortion and sexual health | NA | 86% subjects reported less accessibility to contraception devices; 79% reported increase in risk of gender-based violence; 69% of countries introduced policies to facilitate abortion during pandemic |
| Summaiyya Waseem et al. | 14/11/2020 | J Clin Orthop Trauma. | Scoping Review | NA | Revision of literature on the type of trauma during COVID-19 pandemic | NA | During COVID-19 pandemic: Assault was reduced (US 7.28% to 4.95%); DSH raised (France 2.9% vs 2%); Fall from height increased (UK 6% vs 3%) |
| Bas Tierolf et al. | 17/11/2020 | Child Abuse Negl. | Quantitative and Qualitative Cross-Sectional Study | 87 | Impact of lockdown within families already known to social services | NA | No difference was found in violence between families who participated before and after the lockdown |
| Sawsan Abuhammad | 28/11/2020 | Int J Clin Pract. | Cross-Sectional Study | 687 | Assessment of VAW during pandemic in Jordanian Women | NA | 40% of the participants experienced violence during pandemics. Being married and unemployed were significant predictors of violence |
| Anneen Venter et al. | 04/12/2020 | S Afr Med J. | Retrospective Cross-Sectional Study | NA | Pattern of ED admissions | NA | The decline in the volume of trauma presentations due to IPV was not significant |
| Martina Di Franco et al. | 30/12/2020 | QJM. | Retrospective Cross-Sectional Study | 19160 | Detection of DV during pandemic | WHO Multi-country Study on Women's Health and Domestic Violence against Women Questionnaire | 22.67% disclosed a recent history of domestic violence, after completing the WHO questionnaire. Of those not participating to the survey, diagnosis of domestic violence was only 0.6% |
| Sucharita Maji et al. | 11/01/2021 | J Community Appl Soc Psychol. | Narrative Review | NA | Analysis of DV crisis during COVID-19 pandemics | NA | Major increase in DV cases observed during the covid-19 period, higher during the initial phases of the pandemic then gradually decreased |
| Olufunmilayo I. Fawole et al. | 20/01/2021 | BMC Women's Health | Case Reports | 7 | VAW during Pandemic in Nigeria | NA | Participants reported threats of being forced out of their homes, increasing exposure to COVID-19 and threats regarding custody of children |
| Katherine A. Muldoon et al. | 05/02/2021 | BMC Medicine | Retrospective Cross-Sectional Study | NA | Impact of COVID-19 on violence | NA | Sexual Assault and Domestic Violence Program cases dropped; significant increase in psychological abuse (11.69% in 2018vs 28.57% in 2020) and assaults occurring outdoors (5.19% vs 22.86%) |
| Marwan Akel et al. | 08/03/2021 | J Interpers Violence. | Cross-Sectional Study | 172 | Define pattern of VAW during pandemic | NA | Being a Muslim female and having higher anxiety were significantly associated with higher total abuse scores; Higher stress score in female was significantly associated with lower total abuse scores |
| Akshaya Krishnakumar et al. | 10/03/2021 | Asian J Criminol. | Systematic Review | NA | Factors related to increased DV during pandemic | NA | Sources of motivation in DV perpetrators during lockdown were alcohol and unemployment |
| Cedric Gil-Jardiné et al. | 31/03/2021 | Scand J Trauma Resusc Emerg Med | Retrospective Cross-Sectional Study | 796209 | Trends in content of calls to ED | NA | Calls for stress and anxiety peaked 12 days after lockdown declaration. No increase of calls for violence |
| Solveig Bergman et al. | 08/04/2021 | J Fam Violence. | Cross-Sectional Study | 46 | DV during pandemic | NA | Reduction in number of requests during lockdown, with rates returned to normal during gradual reopening |
| Catherine Porter et al. | 17/04/2021 | SSM Popul Health. | Cross-Sectional Study | 1992 | Evaluate the impact of COVID on physical DV | NA | DV increased in Peru during lockdown. 8.3% of young people experienced an increased in DV during lockdown. No difference was found in rates of violence according to gender. The increase is mostly seen in those who already experienced DV in the past |
| Carmen Vives-Cases et al. | 28/04/2021 | Int J Environ Res Public Health. | Descriptive | NA | VAW in Spain during lockdown | NA | The provinces with the highest 016-calls and Protection Orders rates were those with the highest rate of unemployment |
| Eszter Szilassy et al. | 12/05/2021 | BMC Fam Pract. | Cross-Sectional Study | NA | Evaluation of the use of a designed referral and support system on DV patients | NA | Ongoing study explaining the dynamics of DV referrals and support during the pandemic, implementing a new referral system until September 2021 |
| Laura Castoldi et al. | 10/12/2021 | BMC Emerg Med. | Retrospective Cross-Sectional Study | NA | Patterns of emergency surgery | NA | ED admittance due to DV decreased of 59% |

| MANUAL SEARCH | | | | | | | | | | |  |
| --- | --- | --- | --- | --- | --- | --- | --- | --- | --- | --- | --- |
| Authors | **Date of Publication** | **Journal** | **Study Design** | **Number of Sample** | | | **Tested Outcome** | | **Scales and Measures** | **Main Findings** | |
| Mahmoud K. Al-Omiri et al. | 26/02/2021 | Frontiers in Psychiatry | Cross-Sectional Study | | 1319 | Relationship between Personality Factors and COVID-19 impact on mental health | | NEO Five-Factor Inventory (NEO-FFI); Visual Analog Scale (VAS) | | Higher Neuroticism was found to be associated with more negative COVID19-related changes and impacts. While higher Extraversion, Agreeableness and Conscientiousness were associated with more acceptance to restrictive measures. | |
| Anna M. Gogola et al. | 20/05/2021 | International Journal of Environmental Research and Public Health | Cross-Sectional Study | | 604 | The Dark Triad of personality association with pandemic recommendations and anxiety and depressive symptomatology | | Hospital Acquired Depression Scale (HADS); Dirty Dozen Scale | | Individuals with higher levels of psychopathy tend to disobey new rules, while higher levels of subclinical narcissism are linked to better compliance and anxiety and depressive symptoms | |
| Stephen M. Doerfler et al. | 13/04/2021 | International Journal of Psychology | Cross-Sectional Study | | 294 | Effects of Dark Triad Traits and message framing on risky decision-making during pandemic | | Dirty Dozen Scale | | Psychopathy traits were predictor of risk taking in time of pandemic | |
| Marcin Zajenkowski et al. | 16/06/2020 | Personality and Individual Differencies | Cross-Sectional Study | | 263 | Evaluate compliance to restrictive measures linked to Dark Triad and Big Five Personality Traits | | International Personality Item Pool; Levenson’s Self-report Psychopathy Scale; MACH-IV; Narcissistic Admiration and Rivalry Questionnaire; S8* Scale | | Higher levels of Dark Triad Traits correlate with lower level of compliance to containment measures | |
| Alessio Gori et al. | 19/05/2021 | Plos One | Cross-Sectional Study | | 557 | Identification of patterns related to post traumatic symptoms by considering personality and defensive styles | | Impact of Event Scale Revised (IES-R); Forty Item Defense Style Questionnaire (DSQ-40); Italian Ten Item Personality Inventory (I-TIPI) | | Agreeableness and conscientiousness linked to functional coping mechanism; Neuroticism linked to non-functional coping mechanism | |
| Anahita Shokrkon et al. | 19/05/2021 | Plos One | Cross-Sectional Study | | 1096 | Role of neuroticism and extraversion in mental health during COVID-19 | | Mental Health Continuum Short Form (MHC-SF); BFI-2-S | | Extraversion was found to be positively associated to mental health, while neuroticism related negatively to emotional, psychological and social wellbeing | |

**S2**: Included studies and main extracted data
